# Function of the *Borrelia burgdorferi* FtsH Homolog Is Essential for Viability both *In Vitro* and *In Vivo* and Independent of HflK/C

**DOI:** 10.1128/mBio.00404-16

**Published:** 2016-04-19

**Authors:** Chen-Yi Chu, Philip E. Stewart, Aaron Bestor, Bryan Hansen, Tao Lin, Lihui Gao, Steven J. Norris, Patricia A. Rosa

**Affiliations:** a Laboratory of Zoonotic Pathogens, National Institute of Allergy and Infectious Diseases, National Institutes of Health, Hamilton, Montana, USA; b Research Technologies Branch, Rocky Mountain Laboratories, National Institute of Allergy and Infectious Diseases, National Institutes of Health, Hamilton, Montana, USA; c Department of Pathology and Laboratory Medicine, McGovern Medical School at UTHealth, Houston, Texas, USA

## Abstract

In many bacteria, the FtsH protease and its modulators, HflK and HflC, form a large protein complex that contributes to both membrane protein quality control and regulation of the cellular response to environmental stress. Both activities are crucial to the Lyme disease pathogen *Borrelia burgdorferi*, which depends on membrane functions, such as motility, protein transport, and cell signaling, to respond to rapid changes in its environment. Using an inducible system, we demonstrate that FtsH production is essential for both mouse and tick infectivity and for *in vitro* growth of *B. burgdorferi*. FtsH depletion in *B. burgdorferi* cells resulted in membrane deformation and cell death. Overproduction of the protease did not have any detectable adverse effects on *B. burgdorferi* growth *in vitro*, suggesting that excess FtsH does not proteolytically overwhelm its substrates. In contrast, we did not observe any phenotype for cells lacking the protease modulators HflK and HflC (ΔHflK/C), although we examined morphology, growth rate, growth under stress conditions, and the complete mouse-tick infectious cycle. Our results demonstrate that FtsH provides an essential function in the life cycle of the obligate pathogen *B. burgdorferi* but that HflK and HflC do not detectably affect FtsH function.

## IMPORTANCE

Lyme disease is caused by *Borrelia burgdorferi*, which is maintained in nature in an infectious cycle alternating between small mammals and *Ixodes* ticks. *B. burgdorferi* produces specific membrane proteins to successfully infect and persist in these diverse organisms. We hypothesized that *B. burgdorferi* has a specific mechanism to ensure that membrane proteins are properly folded and biologically active when needed and removed if improperly folded or dysfunctional. Our experiments demonstrate that FtsH, a protease that fulfills this role in other microorganisms, is essential to *B. burgdorferi* viability. Cells depleted of FtsH do not survive in laboratory culture medium and cannot colonize mice or ticks, revealing an absolute requirement for this protease. However, the loss of two potential modulators of FtsH activity, HflK and HflC, does not detectably affect *B. burgdorferi* physiology. Our results provide the groundwork for the identification of FtsH substrates that are critical for the bacterium’s viability.

## INTRODUCTION

The bacterial cytoplasmic membrane and constituent proteins provide a variety of essential functions for the prokaryotic cell, including the selective uptake of nutrients and ions, protein transport, cell signaling, and the export of waste products. To maintain the efficient functioning of the cytoplasmic membrane proteins, the bacterial cell employs chaperones and proteases. Chaperones escort proteins toward their final destinations and aid in proper folding. However, if the protein is misfolded, mistranslated, or damaged, chaperones present the aberrant proteins to proteases for degradation. Without this membrane quality control, abnormal proteins may aggregate and perturb membrane integrity, interfere with normal protein function, or jam the transport machinery of the cytoplasmic membrane.

In eubacteria, mitochondria, and chloroplasts, the protease FtsH fulfills this role and also regulates other proteins by programmed degradation. FtsH, an ATP-dependent protease anchored to the cytoplasmic membrane of bacteria (1, 2), can dislocate proteins from the membrane, unfold them in an ATP-dependent manner, and processively degrade the proteins (reviewed in reference 3). FtsH is essential to the viability of *Escherichia coli*, *Bradyrhizobium japonicum*, and *Helicobacter pylori* (4–6), while in other bacteria, FtsH depletion reduces cell viability under stress conditions or in the stationary phase but is dispensable during growth under normal, physiological conditions (7–9). In addition to membrane protein quality control, FtsH mediates a variety of other cellular functions by controlling degradation of specific substrates. These functions have been more fully characterized in *E. coli*, where some cytoplasmic proteins are also substrates for FtsH degradation, including the heat shock response regulator σ^32^ (also degraded by other proteases) (10) and λcII, a lambda phage regulator contributing to the decision between a lysogenic and a lytic life cycle (11). LpxC, an enzyme necessary for lipid A biosynthesis, is proteolytically controlled by FtsH. Depletion of cellular FtsH levels creates an imbalance between the synthesis of lipopolysaccharides and phospholipids and results in cell death, therefore making FtsH essential to *E. coli* (4). Although the full range of FtsH substrates remains unknown, several membrane protein targets of FtsH have been identified and include SecY, a subunit of the protein translocation machinery (12), KdtA, a transferase involved in oligosaccharide biosynthesis (13), and YccA, a membrane protein of unknown function (14).

Although the regulation and substrate range of FtsH have not been fully elucidated, even in *E. coli*, two modulators of the protease have been identified. The cytoplasmic membrane proteins HflK and HflC (here, HflK/C) form a high-molecular-weight complex with the protease, referred to as the FtsH holoenzyme (12, 15). Several reports indicate that HflK/C acts as a negative regulator of membrane substrates, selectively allowing specific membrane proteins access to the active site chamber of FtsH (11, 12, 14). Although these results are complex and not completely understood, evidence suggests that entry of cytoplasmic substrates into the FtsH protease may occur through a separate process (14). Unlike FtsH, HflK and HflC are not essential to *E. coli* and can be inactivated, with the main observable phenotype being a high frequency of lysogenization by phage λ (12).

All three proteins FtsH, HflK, and HflC, have chromosomally encoded homologs in *Borrelia burgdorferi*, the Lyme disease spirochete (16). *B. burgdorferi* is an obligate zoonotic parasite with limited metabolic capabilities which cycles between *Ixodes* ticks and vertebrate hosts. To survive in such disparate host environments, the spirochete differentially synthesizes various membrane proteins for sensing external stimuli, nutrient acquisition, immune evasion, and as adhesins. In addition, *B. burgdorferi* produces and maintains periplasmic flagella for motility, which are required for tick transmission and to disseminate and persist in the vertebrate host (17, 18). All of these functions are either embedded in or exported across the cytoplasmic membrane; therefore, membrane quality control is likely to be crucial to *B. burgdorferi* survival. Although the mechanism(s) by which *B. burgdorferi* maintains the proper functioning of cytoplasmic membrane proteins has not been examined, a library of transposon mutants identified insertions in the *hflK* and *hflC* homologs (*bb0203* and *bb0204*, respectively) that resulted in reduced murine infectivity (19). Interestingly, only one transposon insertion at the terminus of the *ftsH* homolog (*bb0789*) was isolated, and the authors postulated that the dearth of insertions in and around the *bb0789* locus indicated that the encoded FtsH homolog confers an essential function to *B. burgdorferi*. Recently, Drecktrah and colleagues observed that *ftsH* expression in *B. burgdorferi* was controlled by *B. burgdorderi* Rel (Rel_Bbu_) in response to nutrient starvation, whereas *hflK* and *hflC* transcript levels were not significantly affected (20).

To investigate the contributions of the FtsH, HflK, and HflC homologs to the life cycle of *B. burgdorferi*, we constructed mutants of all three genes and characterized the strains both *in vitro* and *in vivo*. Surprisingly, our results did not identify any phenotype for the ΔHflK/C double mutant, although we examined morphology, growth rate, growth under stress conditions, and the complete mouse-tick infectious cycle. In contrast, however, FtsH was shown to be essential to *B. burgdorferi* survival, both *in vitro* and *in vivo*. Using an inducible system to control *ftsH* expression, *B. burgdorferi* cells depleted of FtsH exhibited arrested cell growth and morphological defects during *in vitro* cultivation. Mouse-tick infection studies with the mutant strain demonstrated that FtsH is also required for survival in both the murine host and the tick vector. These studies indicate that the *B. burgdorferi* FtsH homolog provides an essential function both *in vitro* and *in vivo*, independently of the homologs of the HflK/C modulators.

## RESULTS

### BB0789, BB0203, and BB0204 are *B. burgdorferi* homologs of FtsH, HflK, and HflC, respectively.

*B. burgdorferi* BB0789, BB0203, and BB0204 are annotated as homologs of *E. coli* FtsH, HflK, and HflC in the NCBI database (16). BB0789 shares 50% identity with *E. coli* FtsH by amino acid sequence alignment but exhibits the highest identity within functional domains: ATPase domain (ATP binding Walker A and B motifs and the second region of homology [SRH]) and the Zn^2+^ protease active-site motif (3, 21) (see Fig. S1 in the supplemental material). BB0203 and BB0204 are 52% and 50% similar to *E. coli* HflK and HflC, respectively, with both proteins having 28% identity to their counterparts. The NCBI conserved domain search identifies BB0203 and BB0204 as members of the SPFH (stomatin, prohibitin, flotillin, and HflK/C) superfamily. BB0789, BB0203, and BB0204 are highly conserved in the *B. burgdorferi* sensu lato complex that causes human infection, with 95% to 98% sequence identity.

### *In vitro* phenotypes of the HflK/C mutant are similar to that of the WT.

**FIG 1:**
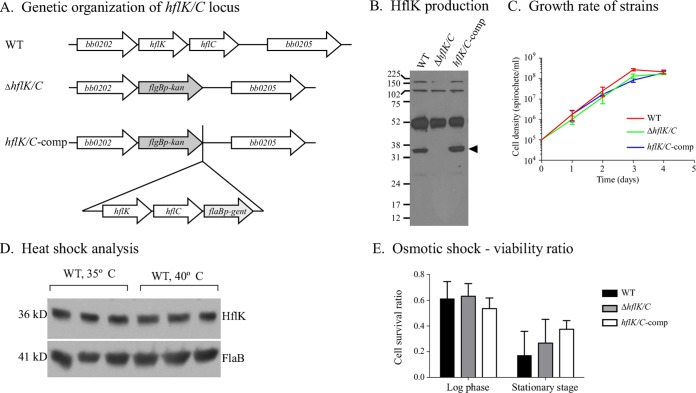
Phenotypic characterization of the Δ*hflK/C* mutant. (A) Genetic organization of the WT, mutant, and complemented strains at the *hflK* and *hflC* loci. (B) Immunoblot analysis of HflK synthesis by different *B. burgdorferi* strains. Antiserum raised against *B. burgdorferi* HflK reacts with a protein of the appropriate size (36 kDa [arrowhead]) from whole-cell lysates of WT and complemented strains, but not with a lysate of the Δ*hflK*/*C* mutant. (C) *In vitro* growth rate of the Δ*hflK*/*C* mutant compared to the WT and *hflK/C-*comp strains. The mean and standard deviation (SD) are displayed. (D) Immunoblot analysis of cell lysates from WT *B. burgdorferi* exposed to a 40°C heat shock treatment for 1 h or maintained at the standard growth temperature (35°C). FlaB levels are shown to demonstrate equivalent protein loads. (E) Cell viability of *B. burgdorferi* strains subjected to 1 N NaCl osmotic stress compared to untreated spirochetes. After treatment, cells were plated in solid BSK medium, and the viability ratio was determined by counting CFU of treated compared to untreated spirochetes. SD bars are shown; no significant difference in cell viability was detected among *B. burgdorferi* strains at either the log phase (one-way analysis of variance [ANOVA], *P* = 0.2850) or stationary phase (one-way ANOVA, *P* = 0.1478).

We hypothesized that FtsH function may be essential to *B. burgdorferi*, based on the lack of transposon insertions observed in a comprehensive signature-tagged mutagenesis library (19). Therefore, we initially focused on the modulators of FtsH substrate specificity, HflK and HflC, homologs of which are widespread among spirochetes. Using an allelic exchange vector, we generated a deletion mutant that lacked both *hflK* and *hflC* (Fig. 1A). The Δ*hflK*/*C* mutant was complemented (*hflK/C*-comp), and the genetic structures of both strains were confirmed by Southern blot and immunoblot analyses (Fig. 1B) (data not shown). Surprisingly, and despite extensive *in vitro* characterization, no distinguishing phenotypes were observed for the Δ*hflK*/*C* strain compared to the wild-type (WT) or complemented strains. The deletion mutant’s growth rate, protein profile (determined by Coomassie-stained SDS-PAGE, and immunoblots reacted with infected mouse sera), plating efficiency, length of time for colony formation on solid medium, and morphology (as assessed by electron microscopy) were similar to those of the WT (Fig. 1C) (data not shown).

In a further attempt to characterize the contribution of HflK/C to *B. burgdorferi* physiology, we assessed the response of the mutant, complemented, and WT strains to the environmental stresses of heat shock and osmotic shock. Previous studies demonstrated that *B. burgdorferi* responds to heat shock by differential production of specific proteins when the cultivation temperature is shifted from 35°C to 39 or 40°C (22–24). However, when we subjected the WT strain to either a 1- or 4-h incubation at 40°C, HflK levels remained unchanged compared to the protein levels from cells cultured at 35°C (Fig. 1D), nor did we see any difference in the relative ability of the mutant to survive a 1-h temperature shift, as assessed by plating for viable spirochetes (data not shown). Finally, we examined the response of the Δ*hflK*/*C* mutant relative to WT and *hflK/C*-comp strains to osmotic stress by exposing cells in different growth phases to 1 N NaCl and then determining cell viability (Fig. 1E). The number of viable spirochetes decreased after salt treatment for each strain at both the log and stationary phases. However, no significant differences in cell viability were detected among *B. burgdorferi* strains at either log phase or stationary phase.

### The Δ*hflK*/*C* mutant persists throughout the *B. burgdorferi* infectious cycle.

**FIG 2:**
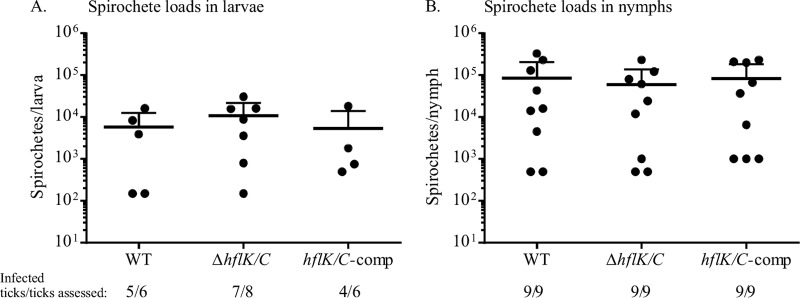
The Δ*hflK/C* strain colonizes ticks as efficiently as WT *B. burgdorferi*. Fed larval *I. scapularis* ticks (A) and nymphs (B) were equally well colonized by all three *B. burgdorferi* strains. Larval ticks were allowed to feed on infected mice, and 8 to 10 days postfeeding, spirochete density was determined by macerating and plating individual ticks and enumerating CFU. Some fed larvae were allowed to molt to the nymphal stage and fed on naive mice, and nymphs were mechanically disrupted 10 days postfeeding and plated to determine spirochete CFU. Each point represents an individual tick, and the mean and upper standard deviation bars are shown (lower SD bars fall below the *x* axis). No significant difference among strains was detected using the Kruskal-Wallis test. The ratios below the graphs denote the number of ticks that had acquired *B. burgdorferi* relative to the number of ticks assessed for each strain.

Although the Δ*hflK*/*C* mutant lacked a distinguishing phenotype *in vitro*, HflK and HflC function might potentially be important *in vivo*. Therefore, we assessed the ability of the mutant, complemented, and WT strains to complete the mouse-tick infectious cycle. However, no defect was observed in the ability to infect mice, either by needle inoculation or via tick bite (Table 1). Further, all three strains infected larval ticks and persisted through the molt at equivalent levels when *Ixodes scapularis* larvae were fed on infected mice, as determined by crushing fed ticks, plating, and counting CFU (Fig. 2). This result was independently confirmed by artificial infection of *I. scapularis* with all three strains and assessment of spirochete loads at both the larval and nymphal stages (data not shown) (25).

**TABLE 1:** Mouse infectivity of the WT, Δ*hflK/C* mutant, and complemented strains via needle inoculation and tick bite

*B. burgdorferi* strain	Infection route^a^	No. of mice with reisolation from tissue/total assessed^b^			No. infected/total
		Ear	Bladder	Joint	
WT	Needle injection	14/14	14/14	14/14	14/14
	Tick bite	2/3	3/3	3/3	3/3
Δ*hflK*/*C* mutant	Needle injection	13/16	13/16	13/16	13/16^c^
	Tick bite	1/3	3/3	3/3	3/3
*hflK*/*C*-comp strain	Needle injection	9/11	9/11	9/11	9/11
	Tick bite	3/3	3/3	3/3	3/3

a The inocula for needle infection were 4 × 10^3^ spirochetes injected intraperitoneally and 1 × 10^3^ spirochetes injected subcutaneously. Cohorts of 5 to 10 infected nymphal ticks were fed on individual mice to assess transmission.

b Data are the results from 3 to 4 independent experiments.

c Mouse infection ratios were not significantly different between groups infected with the Δ*hflK*/*C* and WT strains (unpaired *t* test with Welch’s correction test, *P* = 0.1846).

Subtle defects that might lower the competitive fitness of a strain may not be detected in the gross laboratory assessment of the mouse-tick infectious cycle described above. Two methods of assessing fitness differences between strains were also performed. First, the doses of *in vitro-*grown spirochetes required to infect 50% of inoculated mice (ID_50_) were determined for the Δ*hflK*/*C* mutant (3.16 × 10^2^ spirochetes) and for the WT (2.29 × 10^2^ spirochetes) (see Table S2 in the supplemental material). These values are not significantly different from each other and agreed with the previously determined ID_50_ for WT *B. burgdorferi* strain A3 (26). Finally, mutant and WT strains were coinjected into mice at a 1:1 ratio to determine if the WT had a competitive advantage over the mutant. In three independent trials, each composed of 5 mice, neither strain consistently outperformed the other, as determined by sensitivity to the appropriate antibiotic (see Table S3 in the supplemental material). Further, larval *I. scapularis* ticks were artificially infected by immersion in Barbour-Stoenner-Kelly II (BSKII) medium containing both strains at approximately equivalent densities to determine if the Δ*hflK*/*C* mutant had a reduced-fitness phenotype in the vector. Both strains were present in fed larvae and survived through the molt and subsequent nymph feeding at approximately equal levels, spirochete numbers were quantified by plating crushed ticks on BSKII plates, and the data were analyzed with the Mann-Whitney test (see Fig. S2 in the supplemental material). The WT and Δ*hflK*/*C* mutant were transmitted equally from the infected nymphs to naive mice, as assessed by isolation from mouse tissues (data not shown). These results indicate that inactivation of *bb0203* (*hflK*) and *bb0204* (*hflC*) does not detectably decrease the fitness of *B. burgdorferi* during tick colonization, persistence through the nymphal stage, or transmission to the murine host.

### Generation and characterization of an inducible *ftsH* mutant.

**FIG 3:**
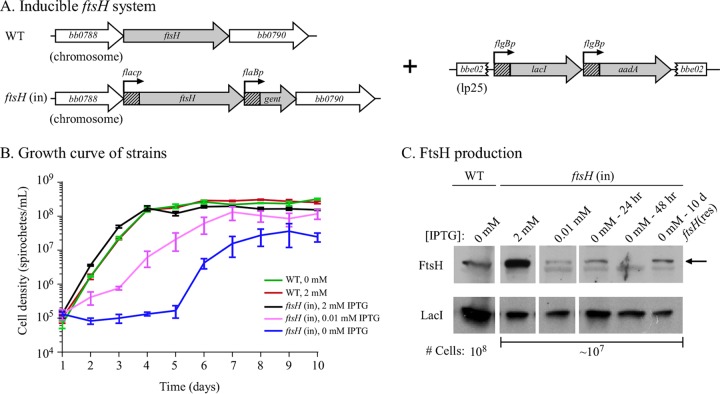
FtsH is required for *B. burgdorferi* cell growth. (A) Genetic organization of an *ftsH*-inducible strain and the corresponding WT strain. The *lacI* repressor was inserted into the *bbe02* locus on linear plasmid lp25 along with the streptomycin-resistance gene (*aadA*). The IPTG-inducible promoter (*flacp*) was derived from the *B. burgdorferi* *flgB* promoter with the 20-nucleotide *lac* operator sequence inserted (27). *flaBp*, *B. burgdorferi* flaB promoter. (B) Growth curve of the *B. burgdorferi* WT (B31-68-LS) and *ftsH*(in) strains. The IPTG-inducible *ftsH*(in) strain demonstrated a growth-dependent response to the concentration of IPTG in the medium. Cell numbers were monitored daily by dark-field microscopy using a Petroff-Hauser counting chamber. (C) FtsH protein levels reflect the IPTG concentration in the *ftsH*(in) strain. An arrow indicates the FtsH protein signal as determined by immunoblot analysis. The IPTG concentration is shown above the immunoblots along with the time post-IPTG depletion. LacI levels are shown to demonstrate relative protein loads. The number of cells loaded in each lane is indicated below the corresponding immunoblots. Note that the immunoblot corresponding to the WT strain contained approximately 10-fold more cell lysate relative to the other immunoblots. The faint band below the FtsH protein is due to nonspecific binding of the antibody to an unrelated protein, as it is present at the same level at all IPTG concentrations.

We attempted to delete *bb0789* (the *ftsH* homolog) from the WT strain, B31-S9, in 6 independent transformations, but were unable to obtain a deletion mutant, supporting the hypothesis that FtsH function is essential to *B. burgdorferi*. Therefore, we generated an inducible *ftsH* mutant using the *lac* promoter/repressor system developed for *B. burgdorferi* (Fig. 3A) (27). Following the example of Gilbert et al., the *lacI* gene and a streptomycin resistance cassette were inserted into the *bbe02* locus of the A3-68 WT strain, and the resulting strain was designated B31-68-LS (27). An allelic exchange construct that fused the inducible promoter, *flacp*, to *ftsH* and was linked to a gentamicin resistance marker was used to replace the endogenous *ftsH* open reading frame, and the resulting strain was designated the *ftsH*(in) strain. The structure of the inducible *ftsH* locus was confirmed by PCR (data not shown), and cell growth was shown to be dependent upon induction (Fig. 3B). Immunoblots confirmed LacI production and isopropyl-β-d-thiogalactopyranoside (IPTG)-controlled synthesis of FtsH (Fig. 3C).

**FIG 4:**
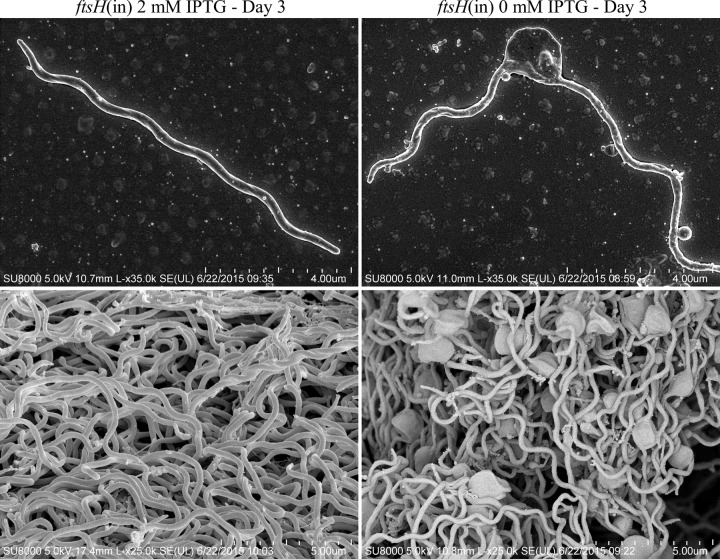
Morphology of FtsH-depleted *B. burgdorferi* cells compared to FtsH^+^ cells. Shown are representative scanning electron micrographs of the *B. burgdorferi* *ftsH*(in) strain grown for 3 days either in the presence of 2 mM IPTG and producing FtsH (left panel) or in the absence of IPTG, resulting in FtsH-depleted cells (right panel). Large membrane blebs are evident in FtsH-depleted cells.

The growth rate of the parental B31-68-LS strain was not affected by the addition of 2 mM IPTG (Fig. 3B). However, the growth rate of the *ftsH*(in) strain was dependent on the concentration of IPTG added to the BSKII medium, growing normally at concentrations ranging from 0.1 mM up to 10 mM (Fig. 3B) (data not shown), but displaying a lower growth rate at or below IPTG concentrations of 0.01 mM. Immunoblot detection of FtsH showed that the *ftsH*(in) strain grown at concentrations of 1 mM IPTG and above produced significantly more FtsH than the parental B31-68-LS (WT) strain (Fig. 3C), but excess FtsH did not seem to adversely affect growth rate. When IPTG was depleted from the *ftsH*(in) culture, cell growth was arrested. Between 48 and 72 h post-IPTG depletion, FtsH levels were undetectable (Fig. 3C), spirochetes became nonmotile, and large membrane distortions developed (Fig. 4). By 72 h postdepletion, >99% of the cells were no longer viable. Strikingly, motile cells were again detected in these cultures around day 6 post-IPTG depletion, and a near-normal growth rate resumed (Fig. 3B). When examined at 10 days post-IPTG depletion, FtsH was again detectable (Fig. 3C), despite the absence of any inducing chemical. We designated this uncloned outgrowth genotype *ftsH*(res), for restored production of FtsH. These results were confirmed in three independent experiments.

**FIG 5:**
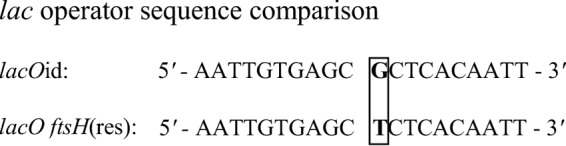
A mutation in the *lac* operator sequence may permit resumption of FtsH production. The ideal *lac* operator sequence (*lacO*id) used for construction of the *ftsH*(in) strain is symmetrical (49). A transversion mutation (boxed) in the *lac* operator isolated from *ftsH*(res) might allow production of FtsH to resume, independent of any inducer, as demonstrated in Fig. 3B and C.

Resumption of FtsH production might theoretically result from a suppressor mutation that eliminates LacI activity or LacI protein or from a mutation in the 20-nucleotide *lac* operator sequence inserted into the normally constitutive *flgB* promoter (27). A deletion or a base mutation in the short operator sequence might decrease the affinity of the LacI repressor for the altered sequence, freeing the promoter to allow transcription and translation to occur, and thereby restoring FtsH production. We tested these hypotheses by examining LacI protein levels from cell lysates obtained at days 2 and 10 post-IPTG depletion. Immunoblots that reacted with an anti-LacI antibody detected constitutive levels of the repressor at both time points, indicating that the suppressor mutation had not reduced or eliminated LacI levels. Sequencing *lacI* from 7 clones isolated from the *ftsH*(res) outgrowth strain did not detect any nucleotide changes from the original *lacI* gene used to transform *B. burgdorferi*, providing further evidence that the nature of the suppressor mutation was not related to LacI activity or production. Therefore, we determined the *lac* operator sequence from 9 clones isolated from the *ftsH*(res) culture. All clones contained a single transversion that altered a G to a T in the *lac* operator sequence (Fig. 5), potentially decreasing the binding affinity of the LacI repressor for the altered sequence.

The requirement for FtsH function *in vitro* indicated that an essential substrate(s) is processed during growth in BSKII medium. *B. burgdorferi* cells lacking FtsH display membrane defects after 48 h and are not viable after 72 h, potentially identifying a window in which FtsH substrates might accumulate and be visible on protein gels. Therefore, we compared WT cell lysate to that of the *ftsH*(in) strain grown in the presence of 1 mM IPTG (overproduction of FtsH) and to that of the *ftsH*(in) strain incubated for 48 h in the absence of inducer (FtsH depleted). However, no discrete differences in protein profiles among samples were observed by either Coomassie-stained or silver-stained gels (data not shown).

### FtsH is required for infection of both mice and ticks.

Although we demonstrated that FtsH is required for *in vitro* survival of *B. burgdorferi*, it is possible that other proteins may be able to compensate for the loss of this protease during infection. To assess the requirement for FtsH *in vivo*, we inoculated mice with the *ftsH*(in) strain cultured *in vitro* with 1 mM IPTG, to ensure FtsH production and cell viability of the inoculum. The *ftsH*(in) strain was unable to establish an infection in mice, nor did the strain survive long enough to elicit an immune response, although all mice inoculated with WT *B. burgdorferi* became infected (Table 2). We also tested the *ftsH*(res) outgrowth strain, which has IPTG-independent expression of FtsH. Interestingly, this strain successfully established infection in 4 of 5 mice and was reisolated from all tissues examined from the 4 infected mice.

**TABLE 2:** Infectivity of the WT, *ftsH*(in), and *ftsH*(res) strains in mice

*B. burgdorferi* strain^a^	No. of mice with reisolation from tissue/total assessed^b^			No. infected/total
	Ear	Bladder	Joint	
A3-68-LS	5/5	5/5	5/5	5/5
*ftsH*(in)	0/5	0/5	0/5	0/5
*ftsH*(res)^b^	5/5	5/5	5/5	5/5

a The inocula for needle infection were 8 × 10^3^ spirochetes injected intraperitoneally and 2 × 10^3^ spirochetes injected subcutaneously.

b Day 10 outgrowth was isolated from the *ftsH*(in) strain cultured in the absence of IPTG and designated the *ftsH*(res) strain. A spontaneous mutation in the *lac* operator sequence presumably allowed for resumption of FtsH production and cell growth.

Since the *ftsH*(in) strain was not able to infect mice, we assessed the strain’s ability to infect and persist in *Ixodes* ticks by artificially infecting larvae by immersion in media containing either the WT or *ftsH*(in) strain. The immersion inocula were plated, and the WT and *ftsH*(*in*) strains were shown to be at similar concentrations: 5.6 × 10^7^ and 5.0 × 10^7^ spirochetes/ml, respectively. Artificially infected larvae were subsequently fed to repletion on naive mice, and spirochete burdens were again assessed directly by mechanical disruption of the ticks in BSK medium and plating in solid medium to enumerate viable colony-forming units. Eight of seventeen ticks (47%) exposed to WT *B. burgdorferi* were infected, and the spirochete burden/infected larvae was 24,600 ± 20,359 (mean ± standard deviation [SD]; *n* = 6). In contrast, none of 17 larvae exposed to the *ftsH*(in) strain contained viable spirochetes after the blood meal. An additional 10 ticks exposed to the *ftsH*(in) strain were assessed for infection after the molt to the nymphal stage, and again, no viable spirochetes were detected. Overall, the data indicate that FtsH provides an essential function necessary for *B. burgdorferi* viability throughout the mouse-tick infectious cycle.

## DISCUSSION

*B. burgdorferi*, an obligate parasite, transitions between arthropod vectors and mammalian hosts, environmental conditions that are substantially different from each other. For example, nutrients, cell density, pH, and temperature all shift between hosts, and many of these cues result in production of different proteins (28–32). To be a successful pathogen, *B. burgdorferi* must respond to these rapid environmental changes by controlling both protein turnover and proper functioning of membrane processes, such as protein and nutrient transport, environmental sensing/signaling, and flagellar motor function. In other bacteria, the FtsH protease contributes to these crucial cellular functions (reviewed in reference 33).

A homolog of FtsH has been identified in *B. burgdorferi* (16) (see Fig. S1 in the supplemental material), but has not been previously studied in this spirochete. Construction and use of an inducible FtsH strain demonstrated that FtsH is essential to *B. burgdorferi* survival during *in vitro* cultivation. When cellular levels of FtsH were depleted, occurring between 24 and 48 h after removal of inducer (Fig. 3C), growth was arrested, and within 72 h, membrane deformations were clearly visible (Fig. 4). Although substrates of the *B. burgdorferi* FtsH have not been identified, the morphology of FtsH-negative cells suggests that a substrate of this protease contributes to membrane architecture. The substrate(s) that requires FtsH processing for *B. burgdorferi* viability presents an intriguing area of research for future investigation.

Surprisingly, viable cells were again detected on day 6 post-IPTG depletion. These cells were producing FtsH, when assessed by immunoblotting on day 10 (Fig. 3C), despite the absence of any inducer. Investigating the nature of the presumptive suppressor mutation in the *ftsH*(res) outgrowth strain, we identified a single-base mutation (a transversion of G to T) in the *lac* operator sequence (Fig. 5). In studies of the wild-type Lac repressor/operator sequence, this single-base mutation at the central nucleotide reduced the LacI binding affinity for the operator by 99% (34). However, the operator sequence constructed by Gilbert et al. and used in this study contained the “ideal” *lac* operator sequence, which lacks the central nucleotide and is perfectly symmetrical (27). We were unable to find any studies that examined repressor binding affinities for mutations in the ideal *lac* operator, but the transversion identified here would reduce the number of bases conferring symmetry. This result reinforces the essential nature of the *B. burgdorferi* FtsH by demonstrating that only cells with a secondary mutation (i.e., allowing FtsH production) are viable.

In another assessment of the requirement for FtsH, mice injected with the *ftsH*(in) strain did not become infected or seroconvert, whereas both the WT and the *ftsH*(res) strain in the outgrowth culture were infectious in mice (Table 2). The combined *in vitro*, murine, and tick infectivity data demonstrate that FtsH is essential to *B. burgdorferi* survival both in liquid culture and throughout the infectious cycle. Further, strict regulation of FtsH production does not seem to be required, at least under the conditions examined in this study, which seems surprising for a protein with an essential role in cell survival. Production of FtsH in the outgrowth culture was enough to restore infectivity, despite the fact that *ftsH* was no longer under the control of its native promoter. Equally surprising was the finding that overproduction of FtsH by increasing IPTG levels above 0.1 mM did not have any detectable adverse effects on *B. burgdorferi* growth *in vitro*, suggesting that excess FtsH does not proteolytically overwhelm its substrates.

In contrast to the FtsH results, we did not detect any phenotype for cells lacking HflK and HflC. In *E. coli*, these proteins act to modulate FtsH substrate recognition (11, 12, 14), and homologs of these accessory proteins are widespread among spirochetes, although not ubiquitous in prokaryotes. Recently, Toledo et al. demonstrated that FtsH, HflK, and HflC are present in membrane lipid rafts of *B. burgdorferi* (35), consistent with previous findings in *E. coli*, where these three proteins form a large, multimeric inner membrane complex (12, 15). Potentially, HflK and HflC regulate the accessibility of minor membrane substrates to the FtsH protease, and the processing of these substrates is not significant enough to present a phenotype in the laboratory setting but in nature would provide a selective advantage. Our ID_50_ results and mouse-tick competition studies indicate this is an unlikely scenario, but we are unable to eliminate this possibility. Alternatively, the *B. burgdorferi* HflK and HflC proteins may have evolved a function different from that of the *E. coli* homologs and perhaps separate from FtsH function, and we have not yet identified the conditions under which these proteins provide a beneficial function in *B. burgdorferi*.

Further investigation to identify the substrates of FtsH should provide insight into the essential nature of this protease and may indicate specific targets that inhibit *B. burgdorferi* viability. Additional characterization of FtsH-HflK-HflC function will undoubtedly be useful in identifying interesting mechanisms of this spirochete’s physiology and obligate pathogenic life cycle.

## MATERIALS AND METHODS

### Ethics statement.

All animal work was performed according to the guidelines of the National Institutes of Health, *Public Health Service Policy on Humane Care and Use of Laboratory Animals* (36), and the United States Institute of Laboratory Animal Resources, National Research Council, *Guide for the Care and Use of Laboratory Animals* (37). Protocols were approved by the Rocky Mountain Laboratories, NIAID, NIH Animal Care and Use Committee. The Rocky Mountain Laboratories are accredited by the International Association for Assessment and Accreditation of Laboratory Animal Care (AAALAC). All efforts to minimize animal suffering were made.

### Bacterial strains and growth conditions.

The bacterial strains used in this study are listed in Table S1 in the supplemental material. *B. burgdorferi* strains were grown in liquid Barbour-Stoenner-Kelly II (BSKII) medium (38, 39) supplemented with 6% rabbit serum (PelFreez Biologicals, Rogers, AZ) and appropriate antibiotics (kanamycin, 200 µg/ml; streptomycin, 50 µg/ml; gentamicin, 40 µg/ml). Cloning vectors were propagated using *E. coli* strain TOP10 (Invitrogen, Carlsbad, CA) or DH5α (New England Biolabs, Ipswich, MA).

### Sequence analysis.

MacVector version 14.0.3 (MacVector, Inc., Apex, NC) was used to align amino acid sequences with ClustalW. The conserved domains of BB0789, BB0203 and BB0204 were searched and analyzed in the CDD database (CDD v3.12), a conserved domain database for the functional annotation of proteins (http://www.ncbi.nlm.nih.gov/Structure/cdd/wrpsb.cgi).

### Generation of BB0203 and BB0789 antisera.

Protein BB0203 lacking the predicted transmembrane helix domain (matching residues 1 to 35 of BB0203 protein) and a 16-amino-acid peptide matching residues 317 to 332 of BB0789 were commercially synthesized (GenScript, Piscataway, NJ). The 16-amino-acid peptide region is relatively well conserved among eubacteria and was originally described by Tomoyasu et al. (1). BB0203-specific and BB0789-specific antisera were generated by immunizing 1-year-old female New Zealand White rabbits with the corresponding protein in the Rocky Mountain Laboratories Animal Unit.

### Construction of mutant strains.

All *B. burgdorferi* transformations were performed similarly: 10 µg of plasmid DNA was electroporated into wild-type strain B31-S9 (or specified strains). All *B. burgdorferi* transformants described were confirmed by PCR and Southern blot analysis, and plasmid content was determined as previously described (40, 41).

All primers used in this study are listed in Table S4 in the supplemental material. The allelic exchange construct for generation of the Δ*hflK*/*C* mutant (Fig. 1A) was constructed by PCR amplification of a 561-bp upstream region of *bb0203* (*hflK*) and a 619-bp downstream region of *bb0204* (*hflC*) from B31-S9 genomic DNA. The DNA fragments were cloned flanking the kanamycin resistance cassette driven by the *B. burgdorferi* *flgB* promoter (42) in pCR-XL-TOPO (Invitrogen). The resulting vector, designated pTAKO.bb0203/0204, was confirmed by sequencing and digestion with the appropriate restriction enzymes.

The mutant Δ*hflK*/*C* strain was complemented by inserting *hflK* and *hflC* into the native chromosomal locus adjacent to the deletion mutation (Fig. 1A). In brief, a DNA fragment containing *hflK* and *hflC* with 232 bp of the putative native promoter region was PCR amplified and sequenced to confirm nucleotide fidelity. This fragment was cloned into the allelic exchange vector (described above) with the gentamicin resistance cassette driven by the *flaB* promoter (43), creating vector pTAComple.bb0203/0204, and inserted after the kanamycin resistance cassette.

We attempted to isolate a deletion mutant of *ftsH* using an approach similar to that described above for the *ΔhflK*/*C* mutant. However, after 6 independent electroporations, we were unable to obtain a deletion mutant of *ftsH*, although this approach worked successfully for obtaining the HflK/C mutant. Therefore, we focused on constructing an FtsH-inducible strain.

The *B. burgdorferi* IPTG-inducible promoter system constructed by Gilbert et al. (27) was used to generate an inducible *ftsH* (*bb0789*) mutant. Constructs pTA*flacp* and pBBE02::*lacI*-Strep^r^ were generously provided by Dan Drecktrah and Scott Samuels (see Table S1 in the supplemental material). Plasmid pBBE02::*lacI*-Strep^r^ was used to construct a LacI-producing strain in an infectious background by transformation into *B. burgdorferi* clone B31 A3-68; the resulting strain was designated B31-68-LS (see Table S1). LacI production in B31-68-LS was confirmed by immunoblot analysis with an antibody raised against LacI (Rockland Immunochemicals, Pottstown, PA). The vector for generating an inducible *bb0789* mutant was constructed by amplifying 646 nucleotides of the 3′ region of *bb0788* from wild-type genomic DNA and cloning it upstream of the inducible promoter in plasmid pTA*flacp*. The *bb0789* coding region was amplified and cloned downstream of the *flac* promoter to generate plasmid pTA*bb0788-flacp-bb0789*. The downstream flanking region (*bb0790*) and selectable marker were assembled in pCR-XL-TOPO. First, the *flaBp*-gentamicin resistance cassette was amplified and cloned into pCR-XL-TOPO. Subsequently, 672 nucleotides of the 5′ region of *bb0790* was inserted downstream of the gentamicin marker to produce plasmid pTAflaBp-gent-bb0790. The *bb0788-flacp-bb0789* fragment from pTAbb0788-flacp-bb0789 was subcloned into plasmid pTAflaBp-gent-bb0790 to generate an allelic exchange vector, pTAindu.bb0789. B31-68-LS was electroporated with pTAindu.bb0789 and selected on solid medium for gentamicin resistance in the presence of 2 mM IPTG to generate the inducible *ftsH*(in) strain (Fig. 3A). Transformants were confirmed by amplifying the gentamicin resistance cassette and the *flacp* promoter upstream of *bb0789*.

The IPTG-dependent growth of the *ftsH*(in) strain was assessed by culture in the presence and absence of IPTG. Spirochetes grown in medium supplemented with 2 mM IPTG and appropriate antibiotics were harvested and washed twice in BSK-H medium (Sigma-Aldrich, Atlanta, GA). Cells were resuspended in fresh BSKII medium to a density of 1 × 10^5^ spirochetes/ml and supplemented with IPTG to final concentrations of 0.01, 0.1, 2, 5, or 10 mM or without IPTG. Growth of the parental strain B31-68-LS was also assessed with either 2 mM IPTG or without IPTG. Cultures were grown in triplicate for statistical accuracy, and the entire growth analysis was repeated independently. Cell densities were monitored every 24 h by dark-field microscopy using a Petroff-Hausser chamber.

To confirm IPTG-dependent regulation of BB0789 in the *ftsH*(in) strain, cell lysates from the growth assays were assessed by immunoblot analysis using an antibody raised against an FtsH peptide (described above). Spirochetes inoculated with 0.01, 0.1, 2, or 10 mM IPTG were harvested when cell densities reached approximately 1 × 10^8^ spirochetes/ml. Cell lysates were prepared for immunoblot analysis using rabbit anti-BB0789 antiserum (1:1,000 dilution) and anti-LacI (rabbit) antibody (1:5,000 dilution).

### Experimental mouse-tick infection studies.

Mouse infection studies were performed with 6- to 8-week-old female RML mice, an outbred strain of Swiss-Webster mice reared at the Rocky Mountain Laboratories breeding facility. Mice were inoculated intraperitoneally (4 × 10^3^ spirochetes) and subcutaneously (1 × 10^3^ spirochetes). The number of injected spirochetes was confirmed by plating an aliquot of the inoculum and determining CFU. Approximately 20 *B. burgdorferi* colonies per strain were screened by PCR to confirm the presence of plasmids lp25, lp28-1, and lp36, which are somewhat unstable *in vitro* but required for *B. burgdorferi* infectivity (41, 44). Mouse infection was assessed at 3 weeks postinjection by mouse seroconversion to *B. burgdorferi* antigens and isolation of spirochetes from mouse tissues (ear, bladder, and ankle joint) in liquid culture.

Approximately 100 to 200 naive *I. scapularis* larvae were fed to repletion on each infected mouse. Acquisition of *B. burgdorferi* by larval ticks was assessed 8 to 10 days postfeeding, and spirochete load was enumerated by mechanical disruption and plating. Basically, individual ticks were placed in a 1.5-ml Eppendorf tube containing 0.5 ml BSKII medium and crushed with a sterile, disposable pestle. After disruption of the tick, an additional 0.5 ml of medium was added, and aliquots were plated in solid BSK medium with appropriate antibiotics. Where relevant, the remaining *I. scapularis* larvae were allowed to molt to nymphs, and cohorts of 5 to 10 nymphs were fed on individual naive mice to assess *B. burgdorferi* transmission. Fed nymphs were mechanically disrupted 10 days postfeeding and plated in solid BSK medium to determine spirochete loads.

The relative fitness of *B. burgdorferi* strains was determined in three independent mouse coinfection studies and also within ticks by artificial infection. The inoculum per mouse was an equal mixture of two strains (i.e., the Δ*hflK*/*C* mutant to WT, or the complemented strain to WT) with 5 × 10^3^ or 1 × 10^4^ spirochetes. An aliquot of the inoculum was plated with or without antibiotic selection to determine the actual ratio of strains. Mouse infection was determined by seroreactivity and confirmed by spirochete isolation from mouse tissues (ear, bladder, and ankle joint) at 4, 8, or 10 weeks postinoculation. The ratio of the two strains in mouse tissues was determined by plating spirochetes in solid BSK medium (with or without antibiotic selection) and enumerating CFU and/or by PCR screening of the *B. burgdorferi* colonies from plates lacking antibiotics.

Artificial tick coinfection was previously described (25, 26). Briefly, about 200 *I. scapularis* larvae were artificially infected by immersion in a *B. burgdorferi* culture. To determine the relative fitnesses of two strains, equal numbers of *ΔhflK*/*C* and wild-type cells or the complemented strain and wild type at a combined density of ~1 × 10^8^ spirochetes/ml were mixed together and used for immersion of ticks. The ratio of each strain in the mixture was determined by plating in solid medium, as described above. Each cohort of artificially infected larvae was fed to repletion on naive RML mice and allowed to molt to nymphs. The nymphs were subsequently fed to repletion on naive mice. The ratio of *B. burgdorferi* strains within ticks was determined by mechanically disrupting the ticks and plating with or without antibiotic selection.

### Determination of ID_50_.

The ID_50_s for B31-S9 and the Δ*hflK*/*C* strain were assessed by inoculating mice with 10-fold serial dilutions from 10^5^ to 10^2^ spirochetes for each strain. Six mice were inoculated per dose. The actual number of injected viable spirochetes was determined by plating a portion of the inoculum and enumerating CFU. Mouse infection was assessed by *B. burgdorferi* isolation from mouse tissues at 4 weeks postinoculation. The ID_50_ value for each strain was calculated according to the method of Reed and Muench (45).

### *In vitro* phenotype analysis of the Δ*hflK*/*C* mutant.

To assess any *in vitro* phenotypes of Δ*hflK/C* strain, the growth rate, morphology, and protein profile of the mutant strain were compared to those of the wild type, B31-S9, and the complemented strain. The growth rate was determined by inoculating triplicate 5-ml BSKII cultures with 1 × 10^5^ spirochetes/ml and incubating the cultures at 35°C. Cell densities were monitored every 24 h by dark-field microscopy using a Petroff-Hausser chamber. Morphological characteristics of *B. burgdorferi* strains were observed by scanning electron microscopy, as previously described (46). The protein profiles and antigenic profiles of all three strains were determined by SDS-PAGE and immunoblot analysis using pooled sera from *B. burgdorferi*-infected mice as previously described (26, 47).

Osmotic shock and heat stress assays were performed similarly to compare the response of the Δ*hflK*/*C* strain to that of the WT and complemented strains. These three strains were individually inoculated in triplicate at an initial concentration of 1 × 10^5^ spirochetes/ml, and each was subdivided into 2 portions when cultures reached a cell density of approximately 5 × 10^7^ spirochetes/ml (mid-log phase) or 2 × 10^8^ spirochetes/ml (stationary phase). For the osmotic shock assay, one set of cultures was treated with 1 N NaCl for 10 min for mid-log-phase cultures or 40 min for stationary-phase cultures, similar to the protocol of Elias et al. (48). Control cultures were supplemented with an equivalent volume of BSK medium. After treatment, spirochetes were immediately plated in solid BSK medium, and CFU were subsequently counted to assess cell viability under salt stress by calculating the ratio of CFU with salt treatment relative to that without treatment. The heat shock assays were set up as described above, except that one set of *B. burgdorferi* cultures was incubated for 1 h at 40°C, a temperature demonstrated to induce a heat shock response in *B. burgdorferi* (22–24), and the other was kept at 35°C for 1 h as a control. After treatment, equivalent numbers of spirochetes were harvested, and cell lysates were prepared for immunoblot analysis using rabbit anti-BB0203 antiserum (1:2,000 dilution). The experiment was repeated with the heat shock treatment extended to 4 h.

## SUPPLEMENTAL MATERIAL

Figure S1  Sequence alignment of *E. coli* and *B. burgdorferi* FtsH homologs. Homologs share 50% identity, and identical residues are indicated in boldface type and shaded. Red boxes mark conserved AAA family motifs relative to the *E. coli* FtsH sequence (21). Walker A and B, ATP binding and hydrolysis motifs; Pore, substrate entry pore; SRH, second region of homology; Arg-finger, arginine finger; Zn^2+^ AS, zinc protease active-site motif; Edge, substrate-binding edge strand. The FtsH homologs are *E. coli* strain K-12 FtsH (CDJ73685.1) and *B. burgdorferi* strain B31 BB0789 (NP_212923.1). Download Figure S1, TIF file, 0.4 MB

Figure S2  Tick coinfection results. Naive larvae were artificially coinfected by immersion in a culture containing the Δ*hflK*/*C* and WT strains (left panel) or the *hflK*/*C*-comp and WT strains (right panel). Ticks were mechanically disrupted in an Eppendorf tube with a disposable pestle and individually plated. Each symbol represents the ratio of mutant or complemented strain to WT strain in an individual tick. The dotted lines indicate the ratio of mutant or complemented strains to the WT strain in the immersion culture. Values above the line indicate a predominance of the mutant or complemented strains, while values below the line indicate a predominance of the WT strain. Bars represent the median and interquartile range of the values. One data point for the unfed nymphs in the complement/WT graph is not shown, as the value, 121, was far outside the range of this figure. No significant difference was observed between groups, as determined by the Mann-Whitney test. Download Figure S2, TIF file, 0.1 MB

Table S1  Bacterial strains and plasmids used in this study. Table S1, DOCX file, 0.1 MB

Table S2  Determination of ID_50_ for the WT and *ΔhflK/C* strains. Table S2, DOCX file, 0.05 MB

Table S3  Mouse coinfection studies. Mice were coinfected with approximately equal numbers of the WT strain and either the Δ*hflK/C* mutant or complemented strain. Mouse infection was initially assessed at about 1 week postinoculation by ear-punch culture and subsequently confirmed by spirochete isolation from mouse tissues (ear, bladder, and ankle joint) at 4, 8, or 10 weeks postinoculation. In three independent experiments, no strain consistently predominated, indicating that no strain had a competitive advantage. Table S3, DOCX file, 0.1 MB

Table S4  Primers used in this study. Table S4, DOCX file, 0.1 MB
