# Erratum for Chu et al., Function of the *Borrelia burgdorferi* FtsH Homolog Is Essential for Viability both *In Vitro* and *In Vivo* and Independent of HflK/C

**DOI:** 10.1128/mBio.02135-16

**Published:** 2016-12-20

**Authors:** Chen-Yi Chu, Philip E. Stewart, Aaron Bestor, Bryan Hansen, Tao Lin, Lihui Gao, Steven J. Norris, Patricia A. Rosa

**Affiliations:** a Laboratory of Zoonotic Pathogens, National Institute of Allergy and Infectious Diseases, National Institutes of Health, Hamilton, Montana, USA; b Research Technologies Branch, Rocky Mountain Laboratories, National Institute of Allergy and Infectious Diseases, National Institutes of Health, Hamilton, Montana, USA; c Department of Pathology and Laboratory Medicine, McGovern Medical School at UTHealth, Houston, Texas, USA

## ERRATUM

Volume 7, no. 2, doi:10.1128/mBio.00404-16, 2016. The present address should have appeared as shown above.

