# Supplementary figures and images for: Function of the Borrelia burgdorferi FtsH Homolog Is Essential for Viability both In Vitro and In Vivo and Independent of HflK/C
Source: mBio. 2016 Apr 19;7(2):e00404-16. doi: 10.1128/mBio.00404-16 (PMC4850261; doi:10.1128/mBio.00404-16)

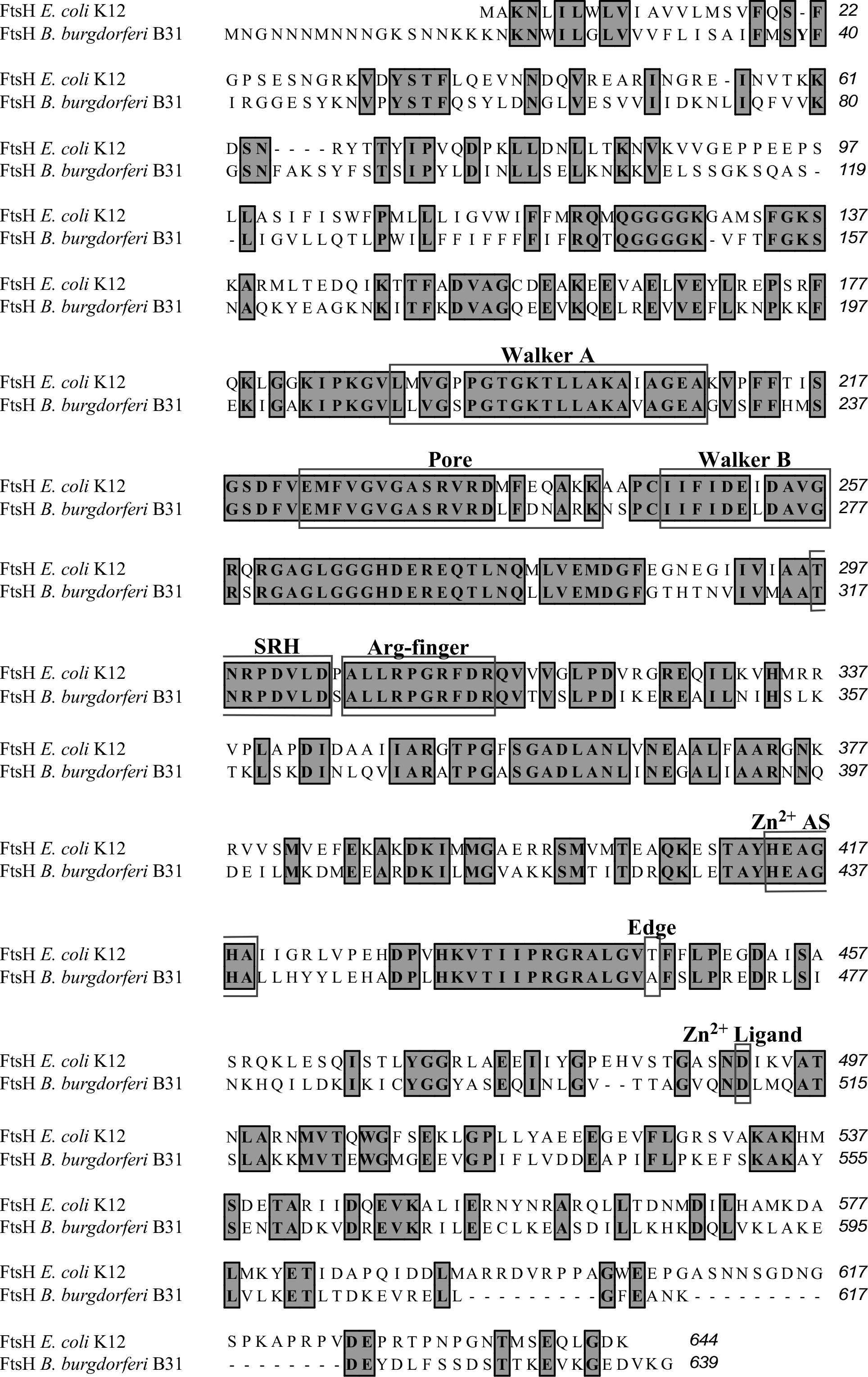

Supplement: Figure S1 — Sequence alignment of E. coli and B. burgdorferi FtsH homologs. Homologs share 50% identity, and identical residues are indicated in boldface type and shaded. Red boxes mark conserved AAA family motifs relative to the E. coli FtsH sequence (21). Walker A and B, ATP binding and hydrolysis motifs; Pore, substrate entry pore; SRH, second region of homology; Arg-finger, arginine finger; Zn2+ AS, zinc protease active-site motif; Edge, substrate-binding edge strand. The FtsH homologs are E. coli strain K-12 FtsH (CDJ73685.1) and B. burgdorferi strain B31 BB0789 (NP_212923.1). Download [file mbo002162785sf1.tif]

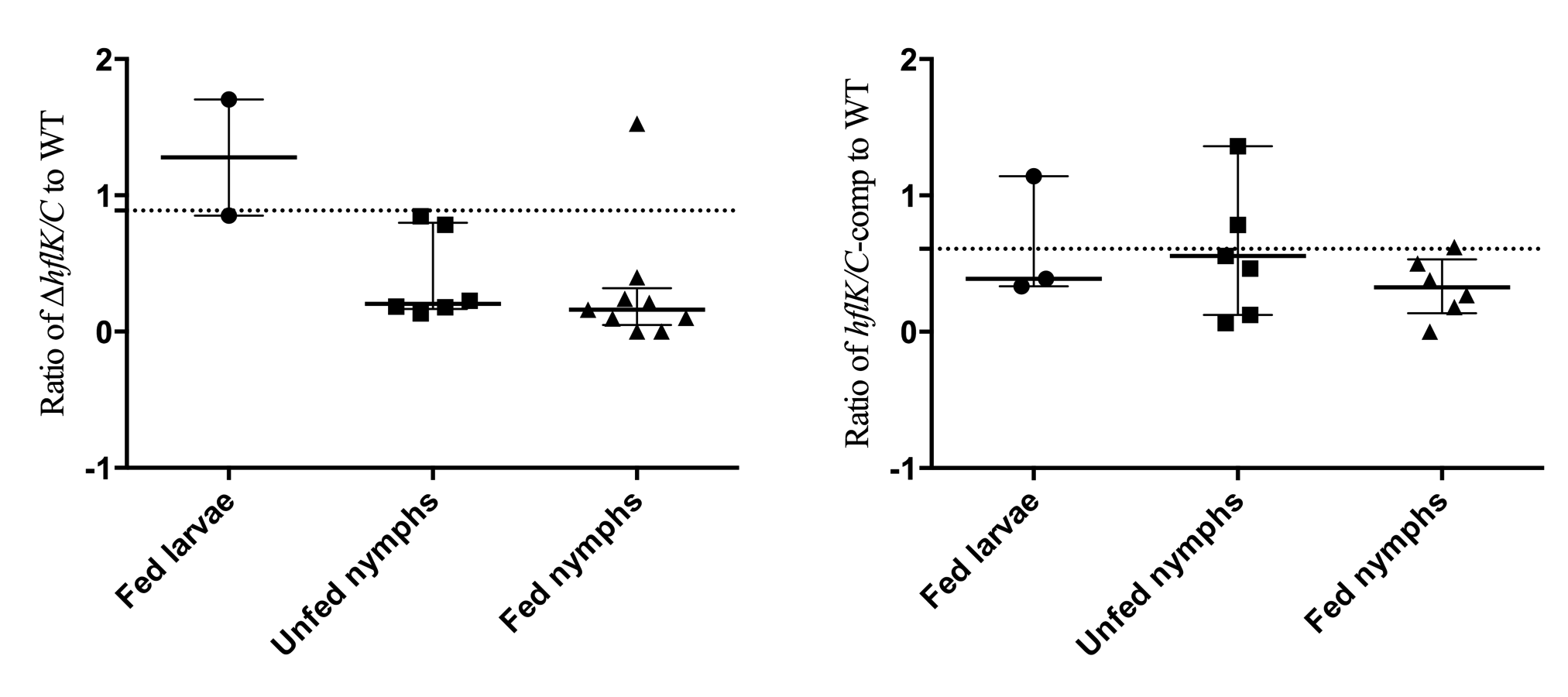

Supplement: Figure S2 — Tick coinfection results. Naive larvae were artificially coinfected by immersion in a culture containing the ΔhflK/C and WT strains (left panel) or the hflK/C-comp and WT strains (right panel). Ticks were mechanically disrupted in an Eppendorf tube with a disposable pestle and individually plated. Each symbol represents the ratio of mutant or complemented strain to WT strain in an individual tick. The dotted lines indicate the ratio of mutant or complemented strains to the WT strain in the immersion culture. Values above the line indicate a predominance of the mutant or complemented strains, while values below the line indicate a predominance of the WT strain. Bars represent the median and interquartile range of the values. One data point for the unfed nymphs in the complement/WT graph is not shown, as the value, 121, was far outside the range of this figure. No significant difference was observed between groups, as determined by the Mann-Whitney test. Download [file mbo002162785sf2.tif]
