# Supplementary material for: Function of the Borrelia burgdorferi FtsH Homolog Is Essential for Viability both In Vitro and In Vivo and Independent of HflK/C
Source: mBio. 2016 Apr 19;7(2):e00404-16. doi: 10.1128/mBio.00404-16 (PMC4850261; doi:10.1128/mBio.00404-16)
Supplement: Table S1 — Bacterial strains and plasmids used in this study. [file mbo002162785st1.docx]

**Table S1.** Bacterial strains and plasmids used in this study

| Strain or plasmid | Description | Reference |
| --- | --- | --- |
| ***B. burgdorferi* strains** |  |  |
| A3-68 | Infectious clone B31-A3 derivative used for construction of *ftsH*(in) strain; contains all plasmids except cp9 and lp56. | (50) |
| B31-68-LS | A3-68 derivative with *bbe02* on lp25 inactivated by insertion of *flgBp::lacI* and *flgBp::aadA* (streptomycin resistance); contains all plasmids except cp9 and lp56. | This study |
| *ftsH*(in) | A3-68-LS derivative harboring *bb0789 (ftsH)* driven by *flacp*; streptomycin and gentamicin resistant; contains all plasmids except cp9 and lp56. | This study |
| *ftsH*(res)  B31-S9 | An outgrowth of the *ftsH*(in) strain obtained 10 days after  depletion of IPTG. This strain incurred a mutation in the lac operator sequence and produces FtsH in the absence of inducer.  A3-68 derivative harboring an inactivated *bbe02* on lp25 and used to generate ∆*hflK/C* strain; streptomycin resistant; contains all plasmids except cp9 and lp56. | This study  (50) |
| Δ*hflK/C* | S9 derivative with *bb0203 (hflK)* and *bb0204 (hflC)* deletion; streptomycin and kanamycin resistant; contains all plasmids except cp9 and lp56. | This study |
| *hflK/C*-comp | Complementation strain restoring *hflK, hflC* and 232 bp of upstream region to Δ*hflK/C;*streptomycin, kanamycin and gentamicin resistant; contains all plasmids except cp9 and lp56. | This study |
| **Plasmids** |  |  |
| pTA*flacp* | pCR2.1-TOPO carrying the inducible *flac* promoter flanked by a 5' AatII and a 3' NdeI site. | (27) |
| pBBE02::*lacI*-Strep^R^ | pCR-XL-TOPO carrying the *flgBp-lacI* fusion and *flgBp-aadA* (spectinomycin resistance) flanked upstream and downstream by *bbe02* sequences. | (27) |
| pTA*bb0788-flacp-bb0789* | pCR2.1-TOPO carrying the inducible *flac* promoter flanked by *ftsH* ORF and 3' region of *bb0788.* | This study |
| pTA*flaBp*-gent-*bb0790* | pCR-XL-TOPO carrying a fusion of *flaBp-aacCI* (gentamicin resistance) and 5' region of *bb0790.* | This study |
| pTAIndu.*bb0789* | Allelic exchange vector for generation of inducible *ftsH* mutant; pCR-XL-TOPO carrying *bb0788*-*flacp*-*ftsH*-*flaBp*-*aacCI*-*bb0790.* | This study |
| pTAKO.bb0203/0204 | Knockout vector of *hflK* and *hflC*; pCR-XL-TOPO carrying *flgBp*-*aphI* (kanamycin resistance) flanked by 561bp upstream of *hflK* and 619bp downstream of *hflC*. | This study |
| pTAComple.bb0203/0204 | Complementation vector derived from pTA KO.bb0203/0204 carrying *hflK,* *hflC,* 232 bp of the putative promoter, and *flaBp-aacC1* (gentamicin-resistance). | This study |
