# Supplementary material for: Function of the Borrelia burgdorferi FtsH Homolog Is Essential for Viability both In Vitro and In Vivo and Independent of HflK/C
Source: mBio. 2016 Apr 19;7(2):e00404-16. doi: 10.1128/mBio.00404-16 (PMC4850261; doi:10.1128/mBio.00404-16)
Supplement: Table S2 — Determination of ID50 for the WT and ΔhflK/C strains. [file mbo002162785st2.docx]

**Table S2.** Determination of ID_50_ for WT and ∆*hflK/C* strains

| Strain |  | # mice infected/# mice inoculated at each inoculum^a^ | |  | Calculated ID_50_^b^ |
| --- | --- | --- | --- | --- | --- |
|  | 10^5^ | 10^4^ | 10^3^ | 10^2^ |  |
| WT | 6/6 | 6/6 | 5/6 | 2/6 | 229 |
| *∆hflK/C* | 6/6 | 6/6 | 5/6 | 1/6 | 316 |

^a^ Mice were injected intra-peritoneal/subcutaneous (80% /20%) with the stated inoculum of each strain in a final volume of 250 μl BSKII. *B. burgdorferi* infection was confirmed by serology and reisolation of spirochetes from the ear, bladder, and joint of each mouse three weeks post-inoculation.

^b^ ID_50_ was calculated following the method of Reed and Muench (Am J Hyg, 1938).
