# Supplementary material for: Function of the Borrelia burgdorferi FtsH Homolog Is Essential for Viability both In Vitro and In Vivo and Independent of HflK/C
Source: mBio. 2016 Apr 19;7(2):e00404-16. doi: 10.1128/mBio.00404-16 (PMC4850261; doi:10.1128/mBio.00404-16)
Supplement: Table S3 — Mouse coinfection studies. Mice were coinfected with approximately equal numbers of the WT strain and either the ΔhflK/C mutant or complemented strain. Mouse infection was initially assessed at about 1 week postinoculation by ear-punch culture and subsequently confirmed by spirochete isolation from mouse tissues (ear, bladder, and ankle joint) at 4, 8, or 10 weeks postinoculation. In three independent experiments, no strain consistently predominated, indicating that no strain had a competitive advantage. [file mbo002162785st3.docx]

**Table S3. Mouse co-infection studies.**

| Mouse coinfection experiment | *B. burgdorferi* strain ^a^ | Mouse ID # | Ratio of Mut (or Comp) to WT ^c^ | | | | |
| --- | --- | --- | --- | --- | --- | --- | --- |
|  |  |  | Inoculum ^b^ | Mouse tissue | | | |
|  |  |  |  | Ear punch | Ear | Bladder | Ankle |
| 1 | WT +Mut M2 | Mouse1 | 1.0 | 0.11 | < 0.003 | < 0.009 | < 0.002 |
|  | WT +Mut M2 | Mouse2 | 1.0 | 1.86 | < 0.002 | < 0.006 | < 0.002 |
|  | WT +Mut M2 | Mouse3 | 1.0 | > 21 | 1.793 | < 0.006 | < 0.005 |
|  | WT +Mut M2 | Mouse4 | 1.0 | > 21 | < 0.005 | < 0.004 | < 0.003 |
|  | WT +Mut M2 | Mouse5 | 1.0 | 0.75 | < 0.014 | < 0.007 | < 0.009 |
| 2 | WT +Mut M2 | Mouse6 | 1.6 | > 91 | > 430 | > 41 | 3.514 |
|  | WT +Mut M2 | Mouse7 | 1.6 | > 191 | 8.8 | 1.6 | > 163 |
|  | WT +Mut M2 | Mouse8 | 1.6 | Uninfected | Uninfected | Uninfected | Uninfected |
|  | WT +Mut M2 | Mouse9 | 1.6 | < 0.013 | < 0.017 | < 0.029 | < 0.007 |
|  | WT +Mut M2 | Mouse10 | 1.6 | > 82 | > 16 | > 62 | > 24 |
|  | WT +Comp C2 | Mouse11 | 0.9 | < 0.032 | < 0.026 | > 41 | 0.851 |
|  | WT +Comp C2 | Mouse12 | 0.9 | 0.214 | 0.011 | 0.5 | 1.333 |
|  | WT +Comp C2 | Mouse13 | 0.9 | Uninfected | Uninfected | Uninfected | Uninfected |
|  | WT +Comp C2 | Mouse14 | 0.9 | 0.006 | 0.979 | 0.011 | > 81 |
|  | WT +Comp C2 | Mouse15 | 0.9 | < 0.008 | < 0.013 | < 0.056 | < 0.059 |
| 3 | WT +Mut M9 | Mouse16 | 1.1 | ND | > 331 | > 618 | > 331 |
|  | WT +Mut M9 | Mouse17 | 1.1 | ND | < 0.01 | 0.17 | 0.16 |
|  | WT +Mut M9 | Mouse18 | 1.1 | ND | 0.37 | < 0.005 | 0.11 |
|  | WT +Mut M9 | Mouse19 | 1.1 | ND | Uninfected | Uninfected | Uninfected |
|  | WT +Mut M9 | Mouse20 | 1.1 | ND | > 478 | > 106 | 9.04 |
|  | WT +Comp C1 | Mouse21 | 0.9 | ND | < 0.009 | < 0.005 | < 0.016 |
|  | WT +Comp C1 | Mouse22 | 0.9 | ND | < 0.017 | < 0.001 | < 0.001 |
|  | WT +Comp C1 | Mouse23 | 0.9 | ND | Uninfected | Uninfected | Uninfected |
|  | WT +Comp C1 | Mouse24 | 0.9 | ND | 10.4 | 10 | > 150 |
|  | WT +Comp C1 | Mouse25 | 0.9 | ND | 3.89 | 1.75 | > 152 |

^a^ WT = wild-type strain B31-S9; Mut M2 and Mut M9 are independently-derived *∆hflK/C* mutant clones; and Comp C2 and Comp C1 are independently-derived complemented clones.

^b^ Inoculum ratio (mutant:WT or complement:WT) was calculated by plating an aliquot of the inoculum with or without antibiotic selection to differentiate between strains.

^c^ The ratio of mutant (or complement) to WT was calculated by plating the spirochetes re-isolated from mouse tissues with or without antibiotic selection to differentiate between strains. If a strain was not re-isolated, a value of 1 was assigned to that strain to represent the lower limit of detection and used to calculate the ratio shown. ND: not done.
