# Supplementary material for: Function of the Borrelia burgdorferi FtsH Homolog Is Essential for Viability both In Vitro and In Vivo and Independent of HflK/C
Source: mBio. 2016 Apr 19;7(2):e00404-16. doi: 10.1128/mBio.00404-16 (PMC4850261; doi:10.1128/mBio.00404-16)
Supplement: Table S4 — Primers used in this study. [file mbo002162785st4.docx]

**Table S4.** Primers used in this study

| Primers | Sequence (5'-3') ^a^ | Function |
| --- | --- | --- |
| UPBB203.F | GCCAAAGACCTCATAGAAGTC | Amplify the region upstream of *hflK* |
| BB203Inv.RC.SalI | gtcgacGTCAAACATCTGCTTCTCC | ” |
| DOWNBB203.RC | GTGCCAAGTTTTGATAAGTTTCC | Amplify the region downstream of *hflC* |
| BB204Inv.F.SalI | gtcgacCTCAACAGACATGGATTTC | ” |
| FlgB.F.SalI | gtcgacTACCCGAGCTTCAAGGAAGATT | Amplify *flgB* promoter and kanamycin-resistance cassette |
| Kan.RC.SalI | gtcgacGAGCTAGCGCCGTCCCGTCAA | ” |
| BB203.Nhel.F | gctagcGGCAATTGGGATATCCATTAAACATAA | Amplify *hflK,* *hflC* and 232 bp upstream |
| BB204.SalI.RC | gtcgacTCAATTTCTTTTGTGAAGATATTGAAA | ” |
| pflaB.SalI.F | gtcgacTGTCTGTCGCCTGTGG | Amplify *flaB* promoter-gentamicin-resistance cassette |
| gent.NheI.RC | gctagcTTAGGTGGCGGTACTTGGGTC | ” |
| BB203.Seq3.F | CAAAAGGAAAAGTTTATGAAGC | Generate *hflK* - *hflC* probe |
| BB204.Seq6.RC | CCTGTTGGGATTCTTTGAGG | ” |
| Kan probe F | GCCATATTCAACGGGAAACGT | Generate kanamycin-resistance cassette probe |
| Kan probe R | GTACGGATAAAATGCTTGATGG | “ |

^a^ Restriction enzyme sites are designated in lower-case letters.
